# Dangers from Turpentine Vapor

**Published:** 1879-12

**Authors:** 


Dangers from Turpentine Vapor.—A Parisian correspondent of an English contemporary writes:

The danger of inhaling the vapor of turpentine has been long known, and its pernicious influence on the health is beyond all doubt, as has been verified in several cases occurring in persons sleeping in newly painted rooms, some of which have even proved fatal. Several theories, more or less plausible, have been propounded to explain the prejudicial effects of the inhalation of these vapors; but, whatever be the correct explanation, there is no doubt of the danger of occupying a room recently painted in which turpentine has been employed, before complete desication has taken place. It was pointed out by the Council of Hygienne, that a sudden death which recently took place in Paris was attributable to this cause, it being shown that it could not be ascribed to the lead which entered into the composition of the paint of the room in which the deceased slept; the lead, being fixed and non-volatile, cannot in these cases be accused of being the offending element.

